# Substantial Increase in Compliance with Saturated Fatty Acid Intake Recommendations after One Year Following the American Heart Association Diet

**DOI:** 10.3390/nu10101486

**Published:** 2018-10-12

**Authors:** Miaomiao Zhao, David Chiriboga, Barbara Olendzki, Bin Xie, Yawen Li, Lisa Jo McGonigal, Ana Maldonado-Contreras, Yunsheng Ma

**Affiliations:** 1 School of Clinical Medicine, Shanghai University of Medicine and Health Sciences, Shanghai 201318, China; zhaomm@sumhs.edu.cn; 2 Division of Preventive and Behavioral Medicine, Department of Quantitative Health Sciences, University of Massachusetts Medical School, Worcester, MA 01655, USA; Barbara.Olendzki@umassmed.edu; 3 Division of Cardiovascular Medicine, Department of Medicine, University of Massachusetts Medical School, Worcester, MA 01655, USA; David.Chiriboga@umassmed.edu; 4 School of Community & Global Health, Claremont Graduate University, Claremont, CA 91711, USA; bin.xie@cgu.edu; 5 School of Social Work, San Diego State University, San Diego, CA 92182, USA; yli@mail.sdsu.edu; 6 Department of Family Medicine and Community Health, University of Massachusetts Medical School, Worcester, MA 01655, USA; LisaJo.McGonigal@umassmemorial.org; 7 Department of Microbiology & Physiological Systems, University of Massachusetts Medical School, Worcester, MA 01655, USA; Ana.Maldonado@umassmed.edu

**Keywords:** fatty acids, metabolic syndrome, American Heart Association (AHA) diet

## Abstract

The American Heart Association (AHA) dietary guidelines recommend 30–35% of energy intake (%E) be from total fat, <7%E from saturated fatty acids (SFA), and <1%E from trans fatty acid (TFA). This study evaluates the effect of AHA dietary counselling on fat intake. Between 2009 and 2014, 119 obese adults with metabolic syndrome (MetS), (71% women, average 52.5 years of age, and 34.9 kg/m^2^ of body mass index), received individual and group counselling on the AHA diet, over a one-year study period. Each participant attended 2 individual sessions (months 1 and 12) and 12 group sessions, at one-month intervals. At baseline and one-year, we collected three random 24-h diet recalls (two weekdays and one weekend day). Fat intake patterns over time were analyzed using paired-*t* test and linear mixed-effect models. There was significant variation on SFA and TFA intake per meal, being highest at dinner, in restaurants, and on weekends. Over the one-year study period, daily intake of total fat, SFA, and TFA decreased by 27%, 37% and 41%, respectively (*p*-value < 0.01, each). Correspondingly, the percentage of participants complying with AHA’s recommendations, increased from 25.2% to 40.2% for total fat (*p*-value = 0.02); from 2.5% to 20.7% for SFA (*p*-value < 0.01); and from 45.4% to 62% for TFA (*p*-value = 0.02). Additionally, SFA intake for all meal types at home decreased significantly (*p*-value < 0.05, each). AHA dietary counselling significantly increased the compliance with AHA dietary guidelines, with an eightfold increase in compliance in SFA intake. Nonetheless, ~80% of our participants still exceeded the recommended SFA intake. Substantial efforts are needed to encourage low-SFA and low-TFA food preparation at home, with strong public health policies to decrease SFA and TFA in restaurants and prepared foods.

## 1. Introduction

Cardiovascular disease (CVD) is the leading cause of death in the United States, and the risk is often exacerbated by a poor diet [1]. Saturated fatty acids (SFA) and trans fatty acids (TFA) have been widely understood to have a negative effect on serum cholesterol profiles, thereby increasing the risk of CVD [2,3,4,5]. Numerous randomized controlled trials [6,7,8] and prospective observational studies [9,10,11,12] have provided strong and consistent evidence that a decrease in dietary SFA reduces the risk of CVD events and all-cause mortality.

Worldwide guidelines have promoted the replacement of SFA with polyunsaturated fatty acids (PUFA), while suggesting that TFA intake should be entirely avoided. The current American Heart Association and American College of Cardiology (AHA/ACC) guidelines suggest a decrease in SFA intake to 5–6% of an individual’s total daily energy intake (%E) for those with elevated low-density lipoprotein cholesterol (LDL-C) concentration [13]. The 2015–2020 US Department of Agriculture Dietary Guidelines for Americans also recommends consuming <10%E from SFA for the general population, as well as the replacement of SFA with unsaturated fats [14]. Similarly, the National Lipid Association Expert Panel strongly recommends a healthy diet low in SFA (<7%E) [15]. However, despite these recommendations, SFA intake remains high in the United States [16].

Metabolic syndrome (MetS) is associated with an increased risk of developing CVD and Type 2 Diabetes Mellitus (T2DM) [17,18], and nearly 35% of US adults and 50% of those aged ≥60 years suffer from MetS in 2011–2012 [19]. Epidemiological studies have demonstrated that higher SFA and TFA intake are modifiable dietary risk factors for MetS [20,21,22,23]. However, current SFA and TFA intake and meal-consumption patterns among MetS patients remain elusive. The purposes of AHA dietary recommendations are to promote a healthy diet and lifestyle, and since 1961 the AHA has suggested a reduction in dietary SFA and TFA in order to reduce the risk of CVD [13]. However, to our knowledge, there has been no research so far aimed at evaluating the effectiveness of dietary counselling, based on the AHA dietary guidelines, in decreasing SFA and TFA intake.

In this study, we investigated the effect of AHA-based dietary counselling on SFA and TFA intake among individuals with MetS. At the same time, we explored changes in SFA and TFA in different meal types, meal location, and day of the week. We hypothesized that the AHA dietary counselling would decrease SFA and TFA intake.

## 2. Materials and Methods

### 2.1. Study Design and Participants

Data of this investigation were from the “Can Do” study, which is a randomized controlled clinical trial (Clinical trial registration: NCT00911885). The purpose of the “Can Do” was to compare the multicomponent AHA dietary guidelines with a single-goal dietary suggestion to increase fiber intake for individuals with MetS. From May 2009 to February 2013, recruitment was conducted by the University of Massachusetts Medical School (UMMS), Worcester, MA. The detailed methodology was described previously [24]. In the “Can Do” study, a total of 240 subjects with MetS were randomized to one of two intervention groups: the AHA diet (*n* = 119) or the high-fiber diet (*n* = 121). Because of the focus of this investigation, we performed analyses using data only from the 119 subjects who participated in the AHA diet arm of the study. The study was conducted in accordance with the Declaration of Helsinki, and the protocol was approved by the Institutional Review Board (IRB) of UMMS. All subjects gave their informed consent for inclusion before they participated in the study. Adverse events during the study were reviewed by the study’s Data Safety and Monitoring Board, and reported to the UMMS IRB.

Participants were eligible for the study if they: (1) were 21–70 years old; (2) had a body mass index (BMI) of 30–40 kg/m^2^ and expressed interested in losing weight; (3) had MetS as defined by having any three of the five abnormal indicators [25]: waist circumference >102 cm for men and 88 cm for women, triglycerides levels ≥150 mg/dL, high-density lipoprotein cholesterol (HDL-C) levels <40 mg/dL for men and <50 mg/dL for women, hypertension with systolic blood pressure ≥130 mmHg or diastolic blood pressure ≥85 mmHg, and fasting glucose concentrations ≥110 mg/dL; (4) were non-smokers; (5) had access to a telephone; (6) were able to give informed consent; and (7) had approval to participate in the study from their primary care physician.

Exclusion criteria included: (1) diabetes diagnosis, or fasting blood sugar ≥120 mg/dL; (2) acute coronary events within 6 months before the study; (3) a woman who was pregnant or lactating; (4) eating disorders; (5) bariatric surgery (6) current participation in a weight-loss program; (7) following other dietary regimens; (8) depression with a high risk of suicide; and (9) intention to move out central Massachusetts during the study period.

### 2.2. American Heart Association Dietary Counselling

Participants were instructed to follow the 2006 AHA dietary guidelines [26]: (1) choosing whole-grain, high-fiber foods (≥30 g/day of dietary fiber); (2) consuming lean animal and vegetable protein foods; (3) consuming vegetables and fruits; (4) eating fish at least twice a week; (5) reducing sugar intake; (6) minimizing sugary drinks; (7) reducing sodium intake; (8) no alcohol intake; (9) obtaining 50–55%E from carbohydrate; (10) obtaining 15–20%E from protein; (11) obtaining 30–35%E from fat; (12) limiting saturated fat to <7%E and trans fat to <1%E; and (13) limiting dietary cholesterol intake to <300 mg/day. Each participant was encouraged to attend 2 individual sessions and 12 group sessions during the one-year study period [27]. Details of the individual and group sessions are displayed in Table 1. Each session was audio-recorded, and a random 10% were selected for counselling fidelity review by an auditor.

### 2.3. Dietary Assessment

Three 24-h dietary recalls (two on weekdays and once on weekend) were performed at both baseline and one-year visits in order to determine the average individual dietary intake (within three weeks of the study visit). Dietary recalls were performed by dietitians who were blinded to the intervention process. Participants were provided with a booklet detailing food portions to estimate portion size prior to the call. Data for SFA, TFA, other selected fatty acids (FA), total energy, and other nutrients were calculated using the Nutritional Data System for Research (NDS-R, 2010–2012, Minneapolis, MN, USA) [28].

### 2.4. Statistical Analyses

Continuous variables were summarized as means (95% confidence interval (CI)), and the comparison between groups was based on student’s *t*-test or analysis of variance (ANOVA); categorical variables were presented as percentage and the comparison between groups were based on chi-squared test. Paired-*t* test was conducted to examine SFA, TFA, and other selected FA intake patterns over time. Linear mixed-effect models were conducted to examine the differences of SFA and TFA intake between baseline and one-year visits, meal type, meal location and meal time, with time point set as a fixed effect and participant identification as a random effect. For analyses of meal location, independent variables included time point, meal type, gender, meal location and the interaction of time point, meal type and meal location. Meanwhile, meal time was assessed, with independent variables that included time point, meal type, gender, meal time and the interaction of time point, meal type and meal time. The %E of SFA and TFA were also analyzed after adjusting for total energy intake. All analyses were conducted using SAS 9.4 (SAS Institute, Cary, NC, USA). Two-side *p* values less than 0.05 indicated statistical significance.

## 3. Results

The present investigation included 119 obese subjects with MetS (BMI: 34.9 kg/m^2^, 95% CI 34.4 to 35.5). The average age was 52.5 years old (95% CI 50.7 to 54.3 years), and 85 of the 119 participants (71%) were women. Mean attendance to AHA dietary counselling was 7.9 sessions (standard deviation (SD) = 3.9) out of a total of 14 sessions. Baseline SFA and TFA intake by demographic characteristics are presented in Table 2. SFA intake was significantly higher in males (*p*-value = 0.001 for g/day) and among Caucasians (*p*-value = 0.038 and *p*-value = 0.020 for g/day and % E). TFA intake was significantly higher in those with lower household income (*p*-value = 0.028 for g/day). During the one-year study, a total of four adverse events were reported. It was determined that the causes of these adverse events were not treatment-related (hysterectomy, pneumonia, lung cancer, and kidney stones).

**Figure 1:**
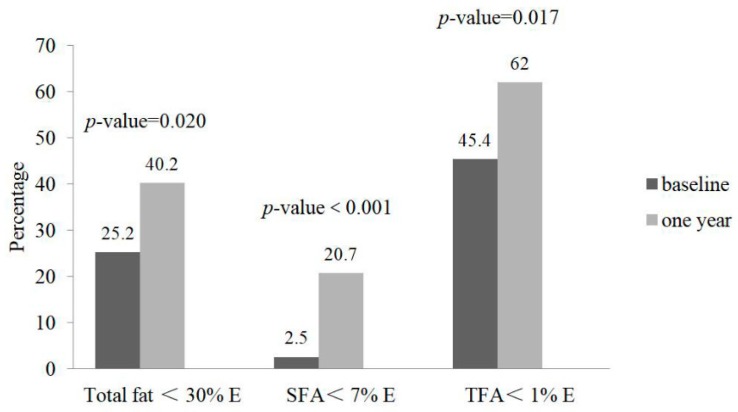
Percentage of participants complying with the AHA dietary intake recommendations for total fat, saturated fatty acid (SFA) and trans fatty acid (TFA) over time.

Figure 1 illustrates the improvement in compliance, between the baseline and the one-year study visit, with the AHA recommendations for total fat, SFA, and TFA intake among study participants. After one-year dietary counselling, percentage of participants who were compliant with the AHA dietary guidelines fat intake recommendations as %E increased from 25.2% to 40.2% for total fat intake (*p*-value = 0.02); from 2.5% to 20.7% (*p*-value < 0.01) for SFA intake; and from 45.4% to 62% (*p*-value = 0.02) for TFA intake.

The comparison of selected daily fat intake (both g and %E) between baseline and one-year follow-up visit are presented in Table 3. Average intake of total fat, SFA and TFA decreased from 74.8 g/day, 26.4 g/day and 2.7 g/day at baseline to 54.4 g/day, 16.7 g/day and 1.6 g/day at one-year (all *p*-values < 0.01); and the corresponding %E decreased from 33.1%, 11.6% and 1.2% to 31.1%, 9.7% and 0.9% (all *p*-values < 0.01). Intake as g/day of total monounsaturated fatty acid (MUFA), oleic acid, total PUFA, linoleic acid (LA), and alpha linolenic acid (ALA) decreased significantly over the one-year period (all *p*-values < 0.05); however, their intakes as %E did not decrease. Only the intake as %E of arachidonic acid (AA) decreased significantly (*p*-value = 0.021). Meanwhile, total energy intake decreased significantly after the one-year AHA counselling (*p*-value < 0.01).

Table 4 and Table 5 demonstrate the SFA and TFA intake by meal type, location, and day of week. Overall, for SFA, subjects consumed less when they ate at home with significant one-year differences in intake observed at breakfast (from 4.54 g to 2.48 g, *p*-value < 0.05), lunch (from 7.53 g to 5.13 g, *p*-value < 0.05), dinner (from 10.05 g to 6.51 g, *p*-value < 0.05), and snack (from 4.03 g to 2.09 g, *p*-value < 0.05). Subjects also consumed significantly less at lunch at one year when they ate in a restaurant or fast food outlet (from 10.24 g to 6.53 g, *p*-value < 0.05). At the same time, SFA intake from weekday breakfast, dinner, and snacks and weekend lunch, dinner, and snacks decreased significantly from baseline to one-year (all *p*-values < 0.05). However, significant decrease of SFA intake as %E was only observed with snacks consumed at home (from 10.37% to 7.95%, *p*-value < 0.05) and away from home (from 11.13% to 7.06%, *p*-value < 0.05) after the one-year counselling.

Compared to baseline, TFA intake after the one-year counselling decreased when subjects ate at home, with significant differences observed for dinner (from 0.90 g to 0.60 g, *p*-value < 0.05) and snacks (from 0.42 g to 0.17 g, *p*-value < 0.05). For meals consumed in a restaurant/fast food chain, TFA intake during lunch decreased significantly at one year (from 1.81 g to 0.95 g, *p*-value < 0.05). At the same time, TFA intake from lunch in weekend days decreased significantly from baseline to one-year (from 1.12 g to 0. 52 g, *p*-value < 0.05). TFA intake as %E from different meal locations and days of week between baseline and one-year visit were similar.

## 4. Discussion

The present study indicates that the proportion of participants complying with the AHA dietary guidelines for intake of total fat, SFA, and TFA significantly increased after the one-year AHA dietary intervention, by 60%, 800% and 36%, respectively. Correspondingly, significant decreases were observed for daily intake of total fat, SFA, and TFA (both in g/day and as %E). Additionally, SFA intake for all meal types at home decreased significantly. Nevertheless, the SFA intake from a large proportion (79.3%) of participants still exceeded recommended limits.

Dietary SFA intake is not only associated with risk of CVD, but also negatively affects bone mineral density [29] and cancer [30,31,32,33,34]. Foods containing a high proportion of saturated fat include animal products such as cream, cheese, butter, other whole milk dairy products and fatty meats. Certain vegetable products also have high saturated fat content, such as coconut oil and palm kernel oil. Many prepared foods are high in saturated fat content as well, such as pizza, dairy desserts, and sausage. While not all SFA are metabolized the same, detrimental effects of diets rich in total SFA have been widely recognized, and national nutritional guidelines have emphasized the need to replace SFA intakes with unsaturated fats. The implementation strategy currently recommended to achieve the reduction of dietary SFA is to shift from food choices high in saturated fat to those high in polyunsaturated and monounsaturated fats [13,14]. However, this is not only about a better choice of oils, but about shifting away from some foods to others that may be quite different in taste, texture, and overall dietary pattern. To achieve meaningful SFA reductions, the country requires strong public health policies providing information on SFA content alerting customers to improve their food choices; in addition to consumer education and agreements with food producers to limit the use of SFA.

Dietary TFA can be naturally derived from ruminant-based meat and dairy products, or artificially through partially hydrogenated oils (PHOs) in the food industry and manufacturing processes [35]. By the late 1990s in the United States, most TFA in the diet (79%) came from PHOs [36]. In our study, the biggest contributors of TFA were meat, poultry, and fish recipes (12.47% at baseline vs. 18.75% at one year), followed by breads, rolls, biscuits, and other related products (10.75% at baseline vs. 10.70% at one year) (data not shown in table). In the United States, nutritional labelling of TFA content became mandatory in 2006 [37]. In 2007, New York City (NYC) became the first in the United States to pass a regulatory restriction on PHO use, targeting restaurants. In June 2015, the US Food and Drug Administration (FDA) announced that PHO use in foods would be phased out of the US market by June 2018, as they were not considered safe for consumption [38], although companies have now been granted an extension to January 2020 [39]. Our study showed that the percentage of participants who complied with AHA dietary recommendations, the daily intake of TFA (both g/day and %E), and TFA intake for dinner and snacks at home, have all improved after the one-year AHA dietary counselling. Education to encourage consumers to further change their dietary patterns and increase healthy cooking at home are needed, and the FDA recommendations to restrict the use of industrially produced TFA in restaurants should be strictly enforced.

PUFAs have usually been shown to be associated with beneficial health effects on CVD. Omega-3 PUFAs have been demonstrated to decrease the production of inflammatory mediators, having a positive effect in obesity, diabetes, and MetS. Moreover, they significantly decrease the appearance of CVD risk factors [40,41,42]. As for MUFAs, cumulative evidence indicates that dietary MUFAs prevent or ameliorate MetS and CVD risk by favorably modulating blood lipids, blood pressure and insulin sensitivity [43,44,45]. Moreover, the majority of epidemiological data favor the cardioprotective activity of dietary MUFAs [43,46]. In the present study, we found that intake in grams of MUFA, oleic acid, total PUFA, LA, ALA significantly decreased after the one-year counselling; however, their intake as %E did not decrease. Only the intake as %E of AA decreased significantly. Therefore, the significant decrease as g/day may be a result of reduced total energy intake. The AHA dietary counselling in our study was based on the 2006 AHA guidelines, which did not include replacing SFA with PUFA, as recommended in the present AHA guideline [47]. Therefore, we did not observe significant change of unsaturated FAs, except AA, after the one-year counselling.

One of the strengths of our study is the use of three 24-h dietary recalls, which offers information on intake from individual foods, being more precise than Food Frequency Questionnaires, which include mostly food groups [48,49]. Additionally, to our knowledge, this is the first study that reports detailed FA intake and evaluates the effect of the AHA dietary counselling among MetS patients. Long-term management of MetS by consumption of a healthy dietary pattern plays an important role in improving health and quality-of-life outcomes. At the same time, we are looking in detail at changes in SFA and TFA in different meal types, meal location, and day of the week, which is an additional strength of our study.

Several limitations should also be considered in light of these results. First, there was no control group without AHA dietary counselling in this investigation, which limited the interpretation of the results. Second, our study was based on the 2006 AHA guidelines, which did not include replacing SFA with PUFA as recommended in the present AHA guidelines. Third, not all SFA generate the same effects. For example, stearic acid has a neutral effect on total cholesterol (TC), LDL-C, and HDL-C, whereas lauric, myristic, and palmitic acids increase TC, LDL-C, and HDL-C, with myristic acid having the most potent hypercholesterolemic effect [50,51,52]; the type of saturated fat found in dairy products may be protective for chronic disease [53,54,55,56]. However, we only calculated the data for total SFA intake, and analyzed the change of total SFA. We decided to focus on the effect of AHA diet on total SFA intake in this manuscript as this is the current dietary recommendation, thus, further research is needed. Lastly, our findings were obtained from obese adults with MetS (BMI between 30 and 40 kg/m^2^) and the sample size is limited, and therefore it is inadequate for generalization to other populations in the United States.

In conclusion, the one-year AHA dietary counselling increased the proportion of participants complying with the AHA guidelines for dietary intake of total fat, TFA and most prominently SFA, which increased eightfold. However, there is still room for considerable improvement, particularly in levels of SFA intake. Actions that further encourage low-SFA and low-TFA food preparation at home, and strong public health policies that decrease SFA and TFA in restaurants and prepared foods are needed.

## Figures and Tables

**Table 1:** American Heart Association (AHA) dietary intervention in the clinical study.

Month/Session	Topic
Month 1/group 1	Orientation/Getting Started with AHA Eating
Month 1/individual visit #1	Individual Consultation
Month 2/group 2	Hunger, Satiety, and Appetite
Month 3/group 3	AHA Meal Planning: Focus on Whole Grains
Month 3/group 4	AHA Meal Planning: Focus on Proteins
Month 4/group 5	AHA Meal Planning: Focus on Vegetables
Month 5/group 6	AHA Meal Planning: Focus on Fats and Oils
Month 6/group 7	AHA Meal Planning: Focus on Fruits
Month 7/group 8	Four keys to AHA Eating Out
Month 8/group 9	The Science of Taste
Month 9/group 10	Motivation and Long-Term Changes
Month 10/group 11	How to Improve Dietary Quality
Month 11/group 12	Savvy Super Marketing: Supermarket Tour
Month 12/individual visit #2	Individual Consultation

**Table 2:** Daily Saturated Fatty Acids (SFA) and Trans Fatty Acids (TFA) intake at baseline by participants’ demographic characteristics.

Variable	*n* (%)	SFA Intake				TFA Intake			
		g/day	*p*-Value	%E	*p*-Value	g/day	*p*-Value	%E	*p*-Value
**Gender**									
Male	34 (28.6)	32.18 (26.35 to 38.00)	0.001	11.86 (10.77 to 12.94)	0.471	3.14 (2.37 to 3.91)	0.078	1.13 (0.98 to 1.28)	0.524
Female	85 (71.4)	24.09 (21.97 to 26.21)		11.44 (10.86 to 12.02)		2.50 (2.17 to 2.84)		1.20 (1.07 to 1.34)	
**Age group**									
20–40	12 (10.1)	32.87 (26.01 to 39.74)	0.296	11.28 (9.88 to 12.68)	0.771	3.41 (1.78 to 5.03)	0.24	1.08 (0.78 to 1.37)	0.424
41–60	35 (29.4)	24.91 (21.59 to 28.24)		11.20 (10.31 to 12.09)		2.44 (2.01 to 2.86)		1.14 (0.94 to 1.35)	
51–60	46 (38.7)	26.26 (21.62 to 30.90)		11.81 (10.87 to 12.74)		2.89 (2.25 to 3.52)		1.29 (1.10 to 1.48)	
61–70	26 (21.8)	25.66 (21.34 to 29.97)		11.74 (10.62 to 12.86)		2.33 (1.86 to 2.81)		1.09 (0.90 to 1.28)	
**Race/ethnicity**									
Caucasian	108 (90.8)	27.17 (24.74 to 29.59)	0.038	11.75 (11.21 to 12.29)	0.020	2.73 (2.39 to 3.06)	0.406	1.20 (1.08 to 1.31)	0.380
Others	11 (9.2)	18.86 (12.57 to 25.16)		9.69 (8.36 to 11.02)		2.26 (0.84 to 3.68)		1.03 (0.74 to 1.33)	
**Highest education level**									
High school diploma or less	13 (11.0)	30.98 (20.35 to 41.62)	0.335	12.47 (10.67 to 14.28)	0.48	2.59 (1.36 to 3.82)	0.873	1.08 (0.72 to 1.43)	0.777
Bachelor’s degree or less	71 (60.2)	26.23 (23.46 to 29.00)		11.49 (10.87 to 12.11)		2.75 (2.35 to 3.16)		1.19 (1.06 to 1.32)	
Graduate/professional	34 (28.8)	24.86 (20.48 to 29.25)		11.43 (10.34 to 12.52)		2.57 (1.92 to 3.23)		1.21 (0.98 to 1.44)	
**Household income**									
$0–$30,000	12 (10.1)	33.45 (22.53 to 44.37)	0.177	11.67 (9.48 to 13.87)	0.385	4.21 (2.08 to 6.33)	0.028	1.32 (0.93 to 1.72)	0.447
$30,000–$50,000	19 (16.0)	26.60 (21.42 to 31.78)		12.34 (10.98 to 13.71)		2.72 (1.99 to 3.45)		1.35 (0.93 to 1.76)	
$50,000–$75,000	20 (16.8)	25.14 (17.08 to 33.20)		11.31 (9.63 to 12.99)		2.46 (1.96 to 2.95)		1.18 (1.01 to 1.36)	
More than $75,000	43 (36.1)	27.13 (23.85 to 30.42)		11.79 (11.03 to 12.55)		2.61 (2.16 to 3.06)		1.14 (0.98 to 1.30)	
Unclear	25 (21.0)	22.61 (18.73 to 26.50)		10.72 (9.80 to 11.65)		2.25 (1.56 to 2.94)		1.05 (0.85 to 1.26)	
**Components of MetS**									
3	58 (48.7)	24.37 (21.49 to 27.25)	0.03	11.26 (10.47 to 12.06)	0.532	2.61 (2.14 to 3.08)	0.497	1.20 (1.04 to 1.35)	0.783
4	41 (34.5)	30.59 (25.62 to 35.57)		11.82 (11.02 to 12.62)		2.93 (2.31 to 3.55)		1.13 (1.00 to 1.27)	
5	20 (16.8)	23.69 (20.31 to 27.08)		11.90 (10.61 to 13.18)		2.40 (1.76 to 3.03)		1.24 (0.87 to 1.60)	

MetS, metabolic syndrome.

**Table 3:** Comparison of intakes of selected fatty acids between baseline and one year.

	Baseline (*n* = 119)	One Year (*n* = 92)	Change (*n* = 92)	*p*-Value
	Mean (95% CI)	Mean (95% CI)	Mean (95% CI)	
**Total energy (kcal/day)**	1957.7 (1829.1 to 2086.3)	1500.9 (1386.8 to 1615.0)	−489.4 (−604.30 to −374.50)	<0.001
**Total Fat**				
g/day	74.76 (68.95 to 80.58)	54.36 (48.89 to 59.83)	−21.95 (−27.50 to −16.39)	<0.001
%E	33.08 (32.06 to 34.09)	31.06 (29.45 to 32.68)	−2.15 (−3.74 to −0.56)	0.009
**SFA, total**				
g/day	26.40 (24.10 to 28.70)	16.69 (15.00 to 18.38)	−10.51 (−12.95 to −8.08)	<0.001
%E	11.56 (11.05 to 12.07)	9.67 (8.99 to 10.34)	−2.06 (−2.84 to −1.29)	<0.001
**TFA, total**				
g/day	2.69 (2.36 to 3.01)	1.58 (1.32 to 1.84)	−1.19 (−1.59 to −0.80)	<0.001
%E	1.18 (1.08 to 1.29)	0.89 (0.79 to 1.00)	−0.32 (−0.47 to −0.16)	<0.001
**MUFA, total**				
g/day	26.30 (24.29 to 28.30)	19.88 (17.75 to 22.01)	−7.01 (−8.96 to −5.07)	<0.001
%E	11.77 (11.29 to 12.24)	11.35 (10.61 to 12.08)	−0.50 (−1.22 to 0.22)	0.17
**Oleic acid**				
g/day	24.52 (22.61 to 26.43)	18.63 (16.59 to 20.67)	−6.49 (−8.35 to −4.64)	<0.001
%E	11.28 (10.76 to 11.81)	10.98 (10.25 to 11.72)	−0.41 (−1.13 to 0.31)	0.259
**PUFA, total**				
g/day	16.01 (14.40 to 17.62)	13.15 (11.41 to 14.88)	−2.90 (−4.53 to −1.27)	0.001
%E	7.05 (6.63 to 7.48)	7.34 (6.73 to 7.94)	0.39 (−0.27 to 1.05)	0.245
**LA**				
g/day	14.05 (12.58 to 15.53)	11.50 (9.89 to 13.12)	−2.62 (−4.12 to −1.13)	0.001
%E	6.32 (5.92 to 6.71)	6.70 (6.10 to 7.30)	0.40 (−0.24 to 1.03)	0.216
**ALA**				
g/day	1.52 (1.36 to 1.67)	1.26 (1.10 to 1.41)	−0.26 (−0.46 to −0.05)	0.014
%E	0.70 (0.64 to 0.76)	0.76 (0.69 to 0.83)	0.06 (−0.04 to 0.17)	0.22
**AA**				
g/day	0.13 (0.12 to 0.15)	0.11 (0.10 to 0 13)	−0.01 (−0.03 to 0.01)	0.308
%E	0.06 (0.06 to 0.07)	0.07 (0.06 to 0.08)	0.01 (0.00 to 0.02)	0.021
**EPA**				
g/day	0.05 (0.03 to 0.07)	0.05 (0.03 to 0.07)	0.00 (−0.02 to 0.02)	0.774
**DPA**				
g/day	0.03 (0.02 to 0.04)	0.02 (0.02 to 0.03)	0.00 (−0.01 to 0.01)	0.855
**DHA**				
g/day	0.12 (0.08 to 0.17)	0.12 (0.08 to 0.17)	0.01 (−0.03 to 0.06)	0.559

SFA, saturated fatty acid; TFA, trans fatty acid; MUFA, monounsaturated fatty acid; PUFA, polyunsaturated fatty acid; LA, linoleic acid; ALA, alpha linolenic acid; AA, arachidonic acid; EPA, eicosapentaenoic acid; DPA, docosapentaenoic acid; DHA, docosahexaenoic acid. The paired-*t* test was used to examine if the difference between baseline and one-year is equal to zero.

**Table 4:** SFA intake by meal type, weekday and location at baseline and one year.

Location	Breakfast		Lunch		Dinner		Snack	
	Baseline	One-Year	Baseline	One-Year	Baseline	One-Year	Baseline	One-Year
At home								
	*n = 123*	*n = 146*	*n = 93*	*n = 91*	*n = 194*	*n = 176*	*n = 346*	*n = 262*
SFA (g)	4.54 (3.49–5.59) ^2^	2.48 (1.50–3.45) ^1,2^	7.53 (6.35–8.72) ^2^	5.13 (3.93–6.33) ^1^	10.05 (9.19–10.91)	6.51 (5.61–7.41) ^1^	4.03 (3.33–4.73) ^2^	2.09 (1.31–2.87) ^1,2^
SFA (%E)	9.63 (7.97–11.28)	7.68 (6.14–9.21)	11.25 (9.36–13.13)	9.80 (7.91–11.69)	10.45 (9.03–11.87)	9.43 (8.00–10.86)	10.37 (9.28–11.46)	7.95 (6.72–9.19) ^1^
Away from home								
	*n = 30*	*n = 24*	*n = 55*	*n = 64*	*n = 7*	*n = 10*	*n = 131*	*n = 109*
SFA (g)	4.82 (2.80–6.84)	3.64 (1.38–5.89)	6.11 (4.60–7.62)	4.35 (2.94–5.76)	7.99 (3.88–12.11)	5.38 (1.93–8.83)	2.83 (1.80–3.86) ^2^	1.54 (0.42–2.67) ^2^
SFA (%E)	11.03 (7.83–14.23)	10.21 (6.62–13.79)	8.54 (6.14–10.93)	7.61 (5.38–9.84)	8.67 (2.11–15.22)	8.63 (3.14–14.13)	11.13 (9.50–12.76)	7.06 (5.27–8.86) ^1^
Restaurant/fast food								
	*n = 15*	*n = 5*	*n = 33*	*n = 23*	*n = 37*	*n = 24*	*n = 21*	*n = 4*
SFA (g)	8.81 (5.98–11.63) ^3^	9.90 (5.05–14.76) ^3^	10.24 (8.31–12.16) ^3^	6.53 (4.21–8.84) ^1,2^	11.63 (9.80–13.45)	10.60 (8.36–12.85) ^3^	5.83 (3.41–8.25) ^2^	5.62 (0.20–11.04)
SFA (%E)	12.75 (8.25–17.24)	11.18 (3.45–18.91)	9.09 (5.99–12.18)	9.55 (5.87–13.24)	9.11 (6.13–12.08)	8.88 (5.26–12.49)	13.72 (9.88–17.56)	14.41 (5.78–23.04)
Weekdays								
	*n = 102*	*n = 124*	*n = 115*	*n = 123*	*n = 148*	*n = 144*	*n = 332*	*n = 266*
SFA (g)	4.63 (3.49–5.76) ^2^	2.58 (1.54–3.62) ^1,2^	6.46 (5.38–7.53) ^2^	5.08 (4.03–6.12) ^2^	9.33 (8.38–10.29)	7.00 (6.03–7.98) ^1^	3.34 (2.64–4.05) ^2^	2.09 (1.32–2.86) ^1,2^
SFA (%E)	9.73 (7.94–11.52)	7.43 (5.79–9.07) ^2^	9.48 (7.78–11.18)	9.16 (7.51–10.80)	10.01 (8.44–11.57)	9.70 (8.14–11.25)	11.08 (9.97–12.19)	7.50 (6.27–8.73) ^2^
Weekend days								
	*n = 66*	*n = 51*	*n = 66*	*n = 55*	*n = 90*	*n = 66*	*n = 166*	*n = 109*
SFA (g)	5.58 (4.20–6.95) ^2^	3.78 (2.22–5.33) ^2^	9.64 (8.26–11.02) ^2,4^	5.18 (3.68–6.68) ^1^	11.73 (10.54–12.92) ^4^	6.91 (5.53–8.29) ^1^	4.71 (3.80–5.63) ^2,4^	1.87 (0.75–2.98) ^1,2^
SFA (%E)	10.83 (8.66–13.01)	9.91 (7.45–12.38)	11.02 (8.80–13.23)	8.66 (6.28–11.05)	10.49 (8.52–12.46)	8.56 (6.36–10.76)	10.04 (8.60–11.49)	8.52 (6.75–10.28)

All values are means (95% CI). SFA intakes per meal and SFA as a percentage of total energy by meal were estimated by LSMEANS of PROC MIXED model in SAS. *n*: The average number of times during the three 24 h given at the study point that participants ate the corresponding meal at the given location/day. “At home”—meals were eaten at home; “Away from home”—meals were eaten at work, school, friend’s home, party, or reception; “Restaurant/fast food”—meals were eaten at a restaurant, cafeteria, fast food chains, take-out, or store. ^1^ *p*-value < 0.05 and *p* values compared differences between baseline and 1 year. ^2^ *p*-value < 0.05 and *p* values compared differences to dinner at the same time point. ^3^ *p*-value < 0.05 and *p* values compared differences between restaurant/fast food and eaten at home at the same time point. ^4^ *p*-value < 0.05 and *p* values compared differences to weekdays.

**Table 5:** TFA intake by meal type, weekday and location at baseline and one year.

Location	Breakfast		Lunch		Dinner		Snack	
	Baseline	One-Year	Baseline	One-Year	Baseline	One-Year	Baseline	One-Year
At home								
	*n = 123*	*n = 146*	*n = 93*	*n = 91*	*n = 194*	*n = 176*	*n = 346*	*n = 262*
TFA (g)	0.36 (0.17–0.56) ^2^	0.21 (0.03–0.39) ^2^	0.68 (0.46–0.90)	0.38 (0.16–0.61)	0.90 (0.75–1.06)	0.60 (0.43–0.76) ^1^	0.42 (0.30–0.54) ^2^	0.17 (0.03–0.30) ^1,2^
TFA (%E)	0.79 (0.54–1.05)	0.68 (0.45–0.92)	1.09 (0.80–1.38)	0.81 (0.51–1.10)	0.83 (0.61–1.05)	0.80 (0.58–1.02)	0.95 (0.78–1.12)	0.94 (0.75–1.13)
Away from home								
	*n = 30*	*n = 24*	*n = 55*	*n = 64*	*n = 7*	*n = 10*	*n = 131*	*n = 109*
TFA (g)	0.40 (0.01–0.78)	0.47 (0.04–0.90)	0.84 (0.55–1.12)	0.52 (0.26–0.79)	1.10 (0.31–1.90)	0.36 (−0.30–1.03)	0.32 (0.13–0.51)	0.11 (−0.10–0.32)
TFA (%E)	0.90 (0.40–1.40)	1.15 (0.60–1.71)	1.00 (0.63–1.37)	0.78 (0.43–1.12)	1.04 (0.02–2.06)	0.43 (−0.43–1.29)	0.96 (0.70–1.22)	0.64 (0.34–0.94)
Restaurant/ fast food								
	*n = 15*	*n = 5*	*n = 33*	*n = 23*	*n = 37*	*n = 24*	*n = 21*	*n = 4*
TFA (g)	1.12 (0.58–1.66) ^3^	1.61 (0.67–2.54) ^3^	1.81 (1.45–2.18) ^3^	0.95 (0.51–1.39) ^1,2,3^	1.47 (1.12–1.81) ^3^	2.10 (1.67–2.53) ^3^	0.60 (0.14–1.06) ^2^	1.51 (0.46–2.55) ^3^
TFA (%E)	1.64 (0.94–2.34) ^3^	1.87 (0.66–3.08)	1.97 (1.49–2.45) ^2,3^	1.21 (0.64–1.78)	1.18 (0.72–1.64)	1.62 (1.06–2.19) ^3^	1.38 (0.79–1.98)	1.55 (0.20–2.90)
Weekdays								
	*n = 102*	*n = 124*	*n = 115*	*n = 123*	*n = 148*	*n = 144*	*n = 332*	*n = 266*
TFA (g)	0.42 (0.20–0.63) ^2^	0.26 (0.07–0.46) ^2^	0.84 (0.63–1.04)	0.52 (0.32–0.71)	0.97 (0.79–1.15)	0.76 (0.57–0.94)	0.36 (0.24–0.49) ^2^	0.17 (0.03–0.31) ^2^
TFA (%E)	0.90 (0.62–1.18)	0.73 (0.47–0.98)	1.18 (0.92–1.44)	0.91 (0.66–1.17)	0.89 (0.65–1.13)	0.86 (0.62–1.10)	0.94 (0.76–1.11)	0.74 (0.54–0.93)
Weekend days								
	*n = 66*	*n = 51*	*n = 66*	*n = 55*	*n = 90*	*n = 66*	*n = 166*	*n = 109*
TFA (g)	0.48 (0.21–0.74) ^2^	0.38 (0.08–0.68) ^2^	1.12 (0.85–1.38)	0.52 (0.23–0.81) ^1^	1.05 (0.82–1.28)	0.78 (0.52–1.05)	0.49 (0.32–0.66) ^2^	0.19 (−0.02–0.39) ^2^
TFA (%E)	0.88 (0.54–1.22)	0.96 (0.58–1.35)	1.25 (0.91–1.60) ^2^	0.72 (0.35–1.09)	0.80 (0.50–1.11)	0.88 (0.54–1.22)	1.09 (0.86–1.32)	1.25 (0.97–1.54) ^4^

All values are means (95% CI). TFA intakes per meal and TFA as a percentage of total energy by meal were estimated by LSMEANS of PROC MIXED model in SAS. *n*: The average number of times during the three 24 h given at the study point that participants ate the corresponding meal at the given location/day. “At home”—meals were eaten at home; “Away from home”—meals were eaten at work, school, friend’s home, party, or reception; “Restaurant/fast food”—meals were eaten at a restaurant, cafeteria, fast food chains, take-out, or store. ^1^ *p*-value < 0.05 and *p* values compared differences between baseline and 1 year. ^2^ *p*-value < 0.05 and *p* values compared differences to dinner at the same time point. ^3^ *p*-value < 0.05 and *p* values compared differences between restaurant/fast food and eaten at home at the same time point. ^4^ *p*-value < 0.05 and *p* values compared differences to weekdays.
